# Stress induced phosphoprotein 1 overexpression controls proliferation, migration and invasion and is associated with poor survival in oral squamous cell carcinoma

**DOI:** 10.3389/fonc.2022.1085917

**Published:** 2023-01-11

**Authors:** Mauricio Rocha Dourado, Amr Elseragy, Bruno Cesar da Costa, Fábio Haach Téo, Gustavo Narvaes Guimarães, Renato Assis Machado, Maija Risteli, Wafa Wahbi, Clarissa Araujo Gurgel Rocha, Lívia Máris Ribeiro Paranaíba, Wilfredo Alejandro González-Arriagada, Sabrina Daniela da Silva, Ana Lucia Carrinho Ayroza Rangel, Marcelo Rocha Marques, Carlos Rossa Junior, Tuula Salo, Ricardo D. Coletta

**Affiliations:** ^1^ Department of Oral Diagnosis, and Graduate Program in Oral Biology, Piracicaba Dental School, University of Campinas, Piracicaba, São Paulo, Brazil; ^2^ Cancer and Translational Medicine Research Unit, Faculty of Medicine, and Medical Research Center, Oulu University Hospital, University of Oulu, Oulu, Finland; ^3^ Department of Biosciences and Graduate Program in Oral Biology, Piracicaba Dental School, University of Campinas, Piracicaba, São Paulo, Brazil; ^4^ Hospital for Rehabilitation of Craniofacial Anomalies, University of São Paulo (HRAC/USP), Bauru, São Paulo, Brazil; ^5^ Department of Oral and Maxillofacial Diseases, Helsinki University Central Hospital, and Translational Immunology Research Program (TRIMM), University of Helsinki, Helsinki, Finland; ^6^ Gonçalo Moniz Institute, Oswaldo Cruz Foundation, Salvador, Bahia, Brazil; ^7^ Federal University of Bahia, Salvador, Bahia, Brazil; ^8^ Center for Biotechnology and Cell Therapy, D’Or Institute for Research and Education (IDOR), Salvador, Brazil; ^9^ Department of Pathology and Parasitology, Institute of Biomedical Sciences, Federal University of Alfenas, Alfenas, Minas Gerais, Brazil; ^10^ Facultad de Odontología, and Centro de Investigación e Innovación Biomédica (CIIB), Universidad de los Andes, Santiago, Chile; ^11^ Lady Davis Institute for Medical Research and Segal Cancer Center, Jewish General Hospital, and Department of Otolaryngology-Head and Neck Surgery, McGill University, Montreal, QC, Canada; ^12^ Department of Oral Pathology and Oral Medicine, Dental School, Western Paranaí State University, Cascavel, Paraná, Brazil; ^13^ Department of Diagnosis and Surgery, School of Dentistry at Araraquara, São Paulo State University (UNESP), Araraquara, São Paulo, Brazil; ^14^ HUSLAB, Department of Pathology, Helsinki University Central Hospital, University of Helsinki, Helsinki, Finland

**Keywords:** oral cancer, STIP1, prognosis, proliferation, invasion, MIR-218-5p

## Abstract

**Objective:**

Although there have been remarkable achievements in the molecular landscape of oral squamous cell carcinoma (OSCC) in recent years, bringing advances in the understanding of its pathogenesis, development and progression, little has been applied in the prognosis and choosing the optimal treatment. In this study, we explored the influence of the stress induced phosphoprotein 1 (STIP1), which is frequently reported to be highly expressed in many cancers, in OSCCs.

**Methods:**

STIP1 expression was assessed in the TCGA database and in two independent cohorts by immunohistochemistry. Knockdown strategy was applied in OSCC cell lines to determine the impact of STIP1 on viability, proliferation, migration and invasion. The zebrafish model was applied for studying tumor formation and metastasis *in vivo*. The association of STIP1 and miR-218-5p was explored by bioinformatics and mimics transfection.

**Results:**

STIP1 was highly expressed in OSCCs and significantly associated with shortened survival and higher risk of recurrence. STIP1 down-regulation decreased proliferation, migration and invasion of tumor cells, and reduced the number of metastases in the Zebrafish model. STIP1 and miR-218-5p were inversely expressed, and the transfection of miR-218-5p mimics into OSCC cells decreased STIP1 levels as well as proliferation, migration and invasion.

**Conclusion:**

Our findings show that STIP1 overexpression, which is inversely associated with miR-218-5p levels, contributes to OSCC aggressiveness by controlling proliferation, migration and invasion and is a determinant of poor prognosis.

## Introduction

Oral squamous cell carcinoma (OSCC) is the most common malignant head and neck tumor, with high prevalence worldwide (1). Despite advancements in multidisciplinary therapy, there has not been significant improvement in the survival of OSCC patients in the last decades. Epidemiologic data shows that OSCC patients have less than a 50% survival rate within 5 years, which is worsen in advanced cases (2, 3). Besides its heterogeneous and aggressive behavior, the lack of accurate tumor markers for choosing the optimal therapy and predicting the risk of recurrence and survival contribute to a high mortality rate (4). In recent years, large-scale studies have compiled a comprehensive catalog of the main cancer-related alterations to overcome those drawbacks, and accurately classify and define more effective therapies.

In two previous studies we have integrated laser capture microdissection and mass spectrometry analysis to characterize the proteome of OSCC cells. In the first study, the proteome of OSCC cells was compared with normal oral epithelial cells (5), whereas in the second we explored the expression profile of OSCC cells during the invasive process (6). Among many misexpressed proteins was the stress induced phosphoprotein 1 (STIP1), also known as HSP70/HSP90 organizing protein (HOP). STIP1 acts in combination with HSP70 and HSP90 proteins as a chaperone that assist the folding and assembling of nascent polypeptides, and the holding, folding or degradation of damaged proteins (7). The 62 kDa protein (543 amino acids) is composed of five major domains, including three tetratricopeptide repeat domains and two domains rich in aspartate and proline, which are capable of interacting with HSP90 and HSP70 during chaperone machine activation (8, 9). Although STIP1 is mainly found in the cytoplasm of the cells in most tissues, it has been detected in extracellular vesicles released by both normal and tumor cells (10–12). Aside from its canonical role of controlling protein folding, the studies are revealing that STIP1 influences numerous other cellular processes including proliferation, apoptosis and invasion (13–15), and STIP1 expression is correlated with chemoresistance (16) and poor outcome of patients with different types of cancers, including breast, liver and ovarian cancer (17–19). Mechanistically, it was showed that STIP1 may activate distinct signaling pathways in the context of the specific cell type and cancer biology, including PI3K/Akt (14), Wnt/β-catenin (20, 21), ERK1/2 (22), JAK2/STAT3 (16, 23) and ALK2-SMAD1/5 (11). The impact of STIP1 in different types of cancers and the pathways activated by its overexpression were extensively revised in a recent study (7). Little is known about the biological regulation of STIP1 in head and neck cancers. High STIP1 expression was associated with shorter overall survival in patients with papillary thyroid carcinoma (24), and STIP1 serological autoantibodies were correlated with early-stage esophageal squamous cell carcinomas (25, 26). Here, we found that STIP1 is overexpressed in OSCCs compared to non-tumor tissues, and its overexpression is an independent prognostic marker for poor outcomes, making it an attractive therapeutic target. We also show that knockdown of STIP1 levels causes decreased proliferation, migration and invasion of OSCC cells, and further demonstrate that miR-218-5p levels is closely correlated to STIP1 and its forced overexpression decreases STIP1 and the phenotypes-related to STIP1 overexpression.

## Material and methods

### Ethics approval and consent to participate

For the research using the human specimens, approval from the ethics review board of each of the hospitals affiliated with the collaborative study was obtained, and the study was approved by Human Research Ethics Committee of the School of Dentistry, University of Campinas (CAAE: 55927322.0.0000.5418). Written informed consent was obtained from participants in compliance with the World Medical Association Declaration of Helsinki, Ethical Principles for Medical Research Involving Human Subjects. Approval for the Zebrafish experiments was granted by the University of Helsinki under the ethical permission (ESAVI/13139/04.10.05/2017) given by the regional state administrative agency.

### Analyses of the cancer genome atlas data and bioinformatics

In the TCGA RNA-Seq data from 314 primary OSCCs and 44 normal adjacent tissues (non-tumor), the levels of STIP1 expression between tumor and non-tumor samples were initially compared. In addition, Kaplan-Meier curves for overall survival (OS), cancer-specific survival (CSS) and disease-free survival (DFS) were constructed based on STIP1 expression levels and patients’ data, and compared applying log-rank test. Using the OSCC samples only, microRNAs in an inverse expression correlation with STIP1 were searched, followed by comparison of the microRNA expression in the tumors and non-tumors. The target prediction programs, miRTarBase (27), TargetScan (28) and miRWalk (29), were used to confirm the microRNAs targeting STIP1 mRNA.

### Patients and clinicopathological data

The cohorts used in this study were recently described by Dourado et al. (30). The cohort 1 was composed of 85 primary OSCCs and matched nonmalignant oral epithelial tissues, and 17 lymph node metastases, included in a tissue microarray (TMA), from patients treated at the Jewish General Hospital, Montreal (Canada), and the cohort 2 was composed of whole tumor sections derived from surgical specimens of 262 primary OSCCs diagnosed and treated in hospitals in Brazil, Chile and Finland. The main clinicopathological characteristics of the patients included in both cohorts are depicted in **Supplementary Table 1.**

### Immunohistochemistry and staining assessment

The immunohistochemical expression of STIP1 was assessed after the methods of Ferreira do Carmo et al. (31), using the rabbit anti-STIP1 antibody (1:3000, HPA039291, Sigma-Aldrich, USA). The reactions were scored by two pathologists, previous calibrated and assessed for inter-observer agreement (Cohen’s Kappa agreement rate of 0.80), using percentage of positive tumor cells (0, 1: 1%–25%, 2: 26%–50%, 3: 51%–75%, and 4:76%–100% staining) and intensity of the staining (0: negative, 1: weak, 2: moderate and 3: strong staining). The samples were categorized in two groups as low expression (<4 points) or high expression (≥4 points).

### Cell lines

The human OSCC cell lines SCC4 (CRL-1624), SCC9 (CRL-1629), SCC15 (CRL-1623), SCC25 (CRL-1628), CAL27 (CRL-2095) (ATCC, Manassas, VA, USA) and HSC3 (JCRB 0623; Osaka National Institute of Health Sciences, Japan) were cultured in DMEM/F12 medium (1:1 mixture of Dulbecco’s modified Eagle’s medium and Ham’s F12 medium, Invitrogen, USA) supplemented with 10% fetal bovine serum (FBS), 400 ng/ml hydrocortisone (Sigma-Aldrich, USA) and a mixture of antibiotic-antimycotic (Invitrogen, USA). The normal human gingival keratinocyte cell line (HGK) was cultured in serum-free and low calcium media containing specific supplements and antibiotics (Gibco’s Keratinocyte-SFM, Invitrogen, USA). The cells were growth at 37°C in a humidified atmosphere of 5% CO_2_. Cells were regularly tested for mycoplasma contamination (MycoAlert^®^ Mycoplasma Detection Kit, Lonza, Switzerland).

### Quantitative reverse transcription-PCR

Total RNA extracted from cell lines using Trizol reagent (Invitrogen, USA) were subjected to first-strand cDNA synthesis with a reverse transcriptase kit (SuperScript™ IV First-Strand Synthesis System, Invitrogen, USA). Expression levels of STIP1 were determined using specific primers and SYBR^®^ Green PCR master mix (Applied Biosystems, USA) in the StepOnePlus Real Time PCR (Applied Biosystems, USA). The primers used for amplification were: STIP1 forward 5’CTGCAAGACTGTCGACCTAAA3’ and reverse 5’TAGGTTCGCTTGGCTTCTTC3’, and cyclophilin A (PPIA) forward 5’GCTTTGGGTCCAGGAATGG3’ and reverse 5’GTTGTCCACAGTCAGCAATGGT3’. The 2^-ΔΔCt^ quantification method was used, with the housekeeping PPIA (cyclophilin A) as the reference gene for data normalization.

### Western blot

After extraction in a lysis buffer containing 10% sucrose, 1% NP-40, 20 mM Tris-HCl (pH 8.0), 137 mM NaCl, 10% glycerol, 2 mM EDTA and a protease inhibitor cocktail (Roche Diagnosis, USA), 20 µg of total protein were resolved in a 10% sodium dodecyl sulphate polyacrylamide gel electrophoresis (SDS-PAGE) under reducing conditions, and then transferred to nitrocellulose membranes. The membranes were incubated with antibodies against STIP1 (1:5000, HPA039291, Sigma-Aldrich, USA) or β-actin (1:50000, clone AC-15; Sigma-Aldrich, USA) for 2 h. After incubating with anti-rabbit (for STIP1) or anti-mouse (for β-actin) peroxidase-conjugated secondary antibodies, the protein bands were detected using enhanced chemiluminescence (ECL) Western Blotting System (GE Healthcare, USA) and signals captured with an Alliance 9.7 instrument (UVITEC, UK).

### Immunofluorescence

Cells cultured in an 8-well culture chamber glass slide were fixed in 70% ethanol for 30 min and incubated for 1 h with anti-STIP1 antibody diluted 1:3000, followed by incubation with secondary anti-IgG conjugated with fluorescein dye (Vector Labs, Burlingame, CA, USA) at dilution 1:100. Cells were mounted with a fluorescent mounting media containing DAPI (Vectashield, Vector Labs) and examined under a photomicroscope equipped with epifluorescence (Leica Microsystems, Wetzlar, Germany). Cells untreated with primary antibodies were used as negative controls.

### STIP1 stable knockdown

HSC3 and SCC9 cells were incubated with control (MISSION^®^ pLKO.1-puro non-Mammalian shRNA Control Transduction Particles, Sigma-Aldrich, USA) or STIP1 (MISSION shRNA Lentiviral Transduction Particles, TCRN0000243096 and TCRN0000243099, Sigma-Aldrich, USA) shRNA lentiviral particles at multiplicity of infection of 2 in culture medium containing 8 μg/ml of polybrene (Sigma-Aldrich, USA) for 8 h. After washing with PBS, cells were cultured in fresh medium for 15 days in the presence of puromycin dihydrochloride (Sigma-Aldrich, USA) to select resistant cells (2 μg/ml for HSC3 cells and 1 μg/ml for SCC9 cells). The efficacy of knockdown was determined by RT-qPCR and western blot.

### Cell viability and apoptosis assay

Viability of cells cultured in 96-well plates at a density of 3,000 cells/well was measured after 24 h with a CellTiter 96^®^ AQueous One Solution Cell Proliferation Assay (Promega, USA) according to manufacturer’s protocol. In independent experiments, cell death was induced with 125 µM hydrogen peroxide for 1 h, followed by assessment of cell viability.

The cells were labeled with annexin V and 7-AAD (BD Biosciences, USA), and analyzed on a flow cytometer equipped with an argon laser (BD Biosciences, USA) for a minimum of 10,000 events for each sample for apoptosis assessment. Apoptotic cells were quantified as the number of annexin V-PE positive and 7-AAD negative cells divided by the total number of cells.

### Proliferation assays

Proliferation assays were based on growth curves and on measuring bromodeoxyuridine (BrdU) incorporation into DNA using a BrdU cell proliferation ELISA kit (Roche Applied Science, USA). For the growth curves, cells were seeded in 96-well plate at 1,000 cells/well, and proliferation was determined every 24 h, up to 96 h, with the CellTiter 96^®^ AQ_ueous_ One Solution Cell Proliferation Assay (Promega, USA).

### Colony formation assay

Cells (500 cells for HSC3 clones and 1,000 cells for SCC9 clones) were plated in 6-well plates and cultured for 10 days for HSC3 cells and 14 days for SCC9 cells. The medium was replaced every 2 days. The cells in colonies were fixed with 4% paraformaldehyde, stained with 1% toluidine blue and quantified using the ImageJ software (NIH, USA).

### Migration assay

Vertical migration assay was performed in 6.5 nm transwell chambers with 8 µm pores (Corning, NY, USA). Briefly, serum starved cells (8x10^4^ cells/well) were plated to the upper chamber in 200 µl of serum-free DMEM/F12, whereas the lower chamber was filled with 500 µl of DMEM/F12 supplemented with 10% FBS. After incubation of 24 h, nonmigratory cells in the upper chamber were gently removed with a cotton swab and cells that migrated to the bottom of the membrane were fixed and stained with a solution of 1% toluidine blue. After elution with 1% SDS solution, absorbance was measured at 650 nm in an ELISA reader (Bio-Rad Laboratories, USA).

### Invasion assays

Invasion assays were based in transwells covered with myogel (32), in the human myoma organotypic culture (33), and in a 3D spheroid assay. For myoma assay, 700,000 cells were added on the top of the myoma disc and cultured for 20 days. The paraffin sections were prepared for immunohistochemistry with monoclonal pan-cytokeratin AE1/AE3 antibody (Dako). Images were acquired with a DM4000B photo microscope connected to a DFC-320 camera using QWin V3 software (Leica Microsystems, Wetzlar, Germany), and quantified using the ImageJ software (NIH, USA).

For the 3D spheroid assay, spheroids (30,000 cells) were constructed by magnetic bioprinting following the manufacturer’s recommendations (NanoShuttle, n3D Biosciences Inc., Greiner Bio-One). Myogel (3 mg/ml) and type I bovine collagen (1 mg/ml, BD Biosciences, USA) were mixed together and 100 µl of solution were gently dispensed into wells of a 96-well plate. After solidification for 4 h at 37°C, the spheroids were collected in 50 µl of the myogel/collagen solution and plated on the top of previous solidified gel. After another 4 h at 37°C, 100 µl of serum-free medium was added, and replaced by fresh one after 48 h. Images of the spheroids were taken immediately and after 5 days of culture. Cancer cell invasion was calculated in each spheroid by the mean of 16 hotspots of invasion, represented by the farthest distance of the cancer cells from the center of the tumor spheroid.

### Cisplatin treatment

Cells cultured in 96-well plates (3,000 cells/well) were incubated with cisplatin (Sigma-Aldrich, USA) diluted directly in the culture medium in concentrations varying from 2.5 to 40 µM. After 24 and 48 h, cell viability was determined with MTS assay (CellTiter 96^®^ Aqueous Solution Cell Proliferation Assay kit, Promega, USA) according to manufacturer’s protocol.

### Zebrafish larvae micro-injection

Two day-old fishes, grown at 28.5°C in an embryonic medium (5 mM NaCl, 0.17 mM KCl, 0.33 mM CaCl_2_ and 0.33 mM MgSO_4_), were dechorionated, anesthetized with 0.04% tricaine, and injected into the perivitelline space with 1,000 cells labeled with CellTrace™ Far Red (Thermo Fisher Scientific, USA). The larvae were then transferred to a 24-well plate with 1 ml fresh embryonic medium and kept at 34°C for 72 h. Dead larvae were excluded, and alive ones were fixed with 10% paraformaldehyde and mounted in Slowfade Gold Antifade Reagent (Thermo Fisher Scientific) on glass slides for imaging. Fish were imaged using a Nikon Perfect Focus System (PFS3) (Nikon, Tokyo, Japan) with 10x magnification from Eclipse Ti-E inverted widefield microscope and the tumor area was measured using ImageJ software (NIH, USA).

### Quantification of miR-218-5p

RNA isolated from HGK, HSC3 and SCC9 cells with the mirVana miRNA isolation kit (Ambion, USA) was converted into specific cDNA derived from mature microRNAs using TaqMan microRNA Reverse Transcription Kit (Applied Biosystems, USA) and quantified using the TaqMan microRNA assay (assay ID 000521, Assay-on-Demand, Applied Biosystems, USA). The small nucleolar RNA RNU48 was used as endogenous control (assay ID Hs04931161_g1, Applied Biosystems, USA). The microRNA relative expression was normalized against endogenous control and HGK cells.

### Transfection of miR-218-5p mimics

Both HSC3 and SCC9 cells were transfected with miR-218-5p mimics (assay ID MC10328) using the RNAiMAX reagent (Invitrogen, USA) as per the manufacturer’s instructions. As control, cells were transfected with an unspecific scramble sequence (Pre-miR Negative Control #1-miR-1, Life Technologies, USA). After 30 h, cells were harvested and subjected to RT-qPCR and western blot for quantification of STIP1 or assessed for proliferation, migration and invasion, as described above.

### Statistical analysis

Chi-squared test was used to analyze the association of STIP1 expression and clinicopathological features, and survival analyses were carried out with both univariate and multivariate Cox regression. In the multivariate analysis, the stepwise approach taking in consideration both clinical and pathological features of tumors was applied. The difference in the STIP1 expression between tumor and non-tumor samples from TCGA was analyzed using the Mann-Whitney U test, whereas the STIP1 immunohistochemical expression among normal, primary and lymph node metastasis was compared with the Kruskal-Wallis test. The receiver operating characteristic (ROC) curve was applied to determine the sensitivity and specificity of STIP1 levels in the discrimination of poor outcome.

All *in vitro* assays were performed at least three times. Mann-Whitney U test or one-way analysis of variance (ANOVA) with *post-hoc* comparisons based on the Tukey’s multiple comparisons test were applied. Correlation analyses were performed using Spearman’s rank test. The level of significance was settled at 5% (p ≤ 0.05).

## Results

### STIP1 is overexpressed in OSCCs and its expression is associated with outcome of patients

**Figure 1:**
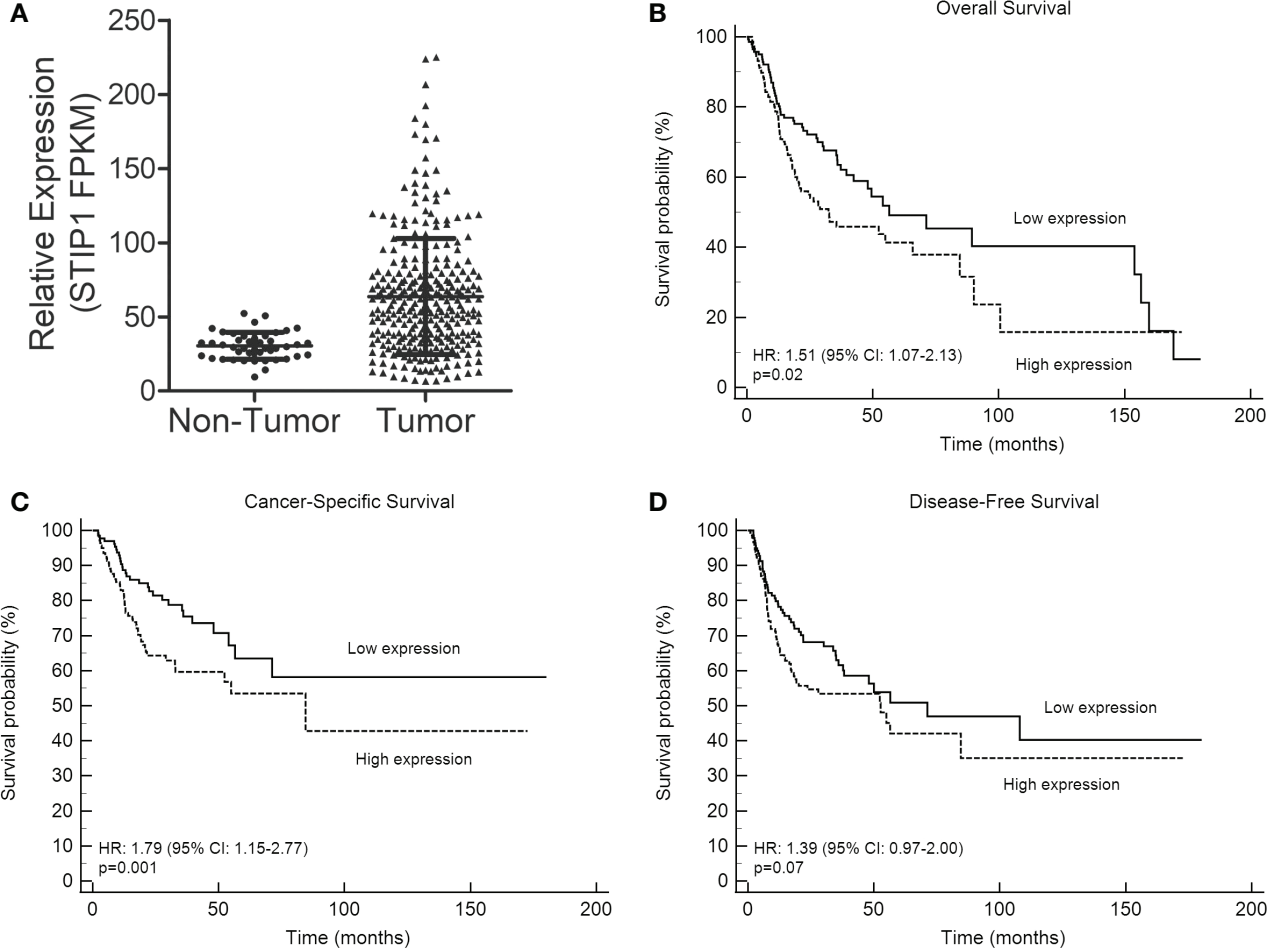
STIP1 is overexpressed in OSCC and its expression levels is associated with outcome. **(A)** Levels of STIP1 in OSCCs and controls (non-tumor) were compared in the RNA-Seq data from the TCGA database, revealing a significantly higher expression in OSCCs (p<0.0001). The impact of STIP1 mRNA levels on overall survival **(B)**, cancer-specific survival **(C)** and disease-free survival **(D)** were assessed based on Kaplan-Meier curves and log-rank test using the TCGA-OSCC cohort.

We initially evaluated the expression of STIP1 in oral cancer and normal oral mucosa (non-tumor) samples from the TCGA database. This analysis revealed that STIP1 mRNA is significantly more expressed in OSCCs compared to non-tumors (p<0.0001, **Figure 1A**). Kaplan-Meier curves based on TCGA-OSCC sample divided by STIP1 levels (with fpkm median value being applied to split the samples in low and high expression levels) revealed a statistically poorer OS (p=0.01) and CSS (p=0.001) and a strong tendency to shortened DFS (p=0.07) for patients classified with high STIP1 expression (**Figures 1B–D**).

Next, using a TMA containing normal oral mucosa, primary OSCCs and OSCC-lymph node metastases (cohort 1), we explored the immunohistochemical expression of STIP1. Immunostaining for STIP1 showed a cytoplasmic pattern, with weak staining restricted to the lower layers of the normal epithelium, whereas tumor cells, in both primary lesions and lymph node metastases, showed variable distribution and intensity (**Supplementary Figure 1**). Quantification of the immunostaining showed higher STIP1 expression in primary tumors (p<0.001) and lymph node metastases (p<0.001) compared to in normal tissues (**Supplementary Figure 1**). Using the data from the 85 OSCC patients included in this cohort, we assessed the association between STIP1 expression and outcome of patients. Kaplan-Meier curves for both CSS and DFS are depicted on **Supplementary Figure 2**. The high immunoreactivity for STIP1 was a marker of reduced CSS with a 5-year survival of 72.1% for the patients with low positivity for STIP1 compared with 52.9% for those with high STIP1 expression, yielding a HR of 2.10 (95% CI: 1.03-4.27, p=0.04) (**Table 1**). As in this cohort clinical stage was also associated with CSS (HR: 2.46, 95% CI: 1.23-4.92, p=0.01) and high STIP1 expression was more frequently detected in patients with advanced clinical stage (p=0.025, **Supplementary Table 2**), we performed multivariate analysis. This analysis revealed that high STIP1 immunopositivity with a HR of 2.56 (95% IC: 1.23-5.36, p=0.012) remained as an independent prognostic factor for OSCC patients, as well clinical stage (HR: 2.14, 95% CI: 1.14-3.99, p=0.017). High STIP1 immunoreactivity showed a tendency towards association with poor DFS in this cohort (p=0.056, **Table 1**).

**Table 1:** Cancer-specific survival and disease-free survival of 85 cases of oral squamous cell carcinoma (cohort 1) based on univariate analysis.

	Cancer-specific survival			Disease-free survival		
	% in 5 years	HR (95% CI)	p value	% in 5 years	HR (95% CI)	p value
Age (years)						
≤ 63 years	66.2	1		56.5	1	
> 63 years	64.2	1.12 (0.57-2.21)	0.73	45.1	1.43 (0.76-2.68)	0.26
Gender						
Male	67.4	1		53.2	1	
Female	66.4	0.92 (0.46-1.81)	0.80	41.0	1.33 (0.70-2.49)	0.36
Clinical stage (7^th^ ed.)						
Early (I + II)	77.8	1		56.7	1	
Advanced (III + IV)	53.9	2.46 (1.23-4.92)	0.01	40.4	1.50 (0.78-2.89)	0.22
Tumor site						
Tongue	61.0	1		51.5	1	
Floor of mouth	75.0	0.50 (0.16-1.58)		60.0	0.95 (0.31-2.94)	
Other	71.8	0.69 (0.2-1.48)	0.47	69.5	0.62 (031-1.23)	0.44
Histopathological grading						
Well-differentiated	90.0	1		68.6	1	
Moderately-differentiated	64.0	1.94 (0.79-4.71)		52.5	1.42 (1.12-5.24)	
Poorly-differentiated	43.8	3.67 (1.25-10.7)	0.05	40.7	3.21 (1.26-8.20)	0.10
Treatment						
Surgery	59.0	1		50.4	1	
Surgery + Radiotherapy	77.3	0.53 (0.25-1.09)		52.6	0.84 (0.23-5.27)	
Surgery + Radiotherapy + Chemotherapy	60.0	0.82 (0.18-2.83)	0.28	44.4	1.62 (0.55-4.75)	0.17
Margin status						
≥5 mm	70.7	1		50.2	1	
<5 mm	64.4	1.14 (0.50-2.58)	0.75	43.6	1.36 (0.66-2.78)	0.39
STIP1						
Low expression	72.1	1		52.4	1	
High expression	52.9	2.10 (1.03-4.27)	0.04	33.0	1.89 (0.99-3.61)	0.056

To strength STIP1 prognostic role in outcomes of patients, we explored the immunoexpression of STIP1 in a larger and multicenter sample composed of 262 patients with OSCC (cohort 2). The pattern of STIP1 staining was quite similar to that performed in the TMA, and representative images for STIP1 in tumors classified as having low and high expression are illustrated in **Supplementary Figure 3**. The Kaplan-Meier curves for CSS and DFS in this cohort is displayed in **Supplementary Figure 4**. High expression of STIP1 was significantly associated with poor CSS (HR: 3.07, 95% CI: 2.04-4.64, p<0.0001) and DFS (HR: 2.19, 95% CI: 1.36-3.53, p=0.003) (**Table 2**). As shown in **Supplementary Table 3**, the high level of STIP1 in OSCCs was significantly associated with age, clinical stage, localization, status of the surgical margins and recurrence. Multivariate analysis was applied to further evaluate the independency of STIP1 expression on the outcomes, revealing STIP1 as an independent marker for both CSS (HR: 3.08, 95% CI: 2.06-4.60, p<0.0001) and DFS (HR: 1.93, 95% CI: 1.27-2.93, p=0.02).

**Table 2:** Univariate analysis for cancer-specific survival and disease-free survival of the patients with oral squamous cell carcinoma of cohort 2 (n=262).

	Cancer-specific survival			Disease-free survival		
	% in 5 years	HR (95% CI)	p value	% in 5 years	HR (95% CI)	p value
Age						
≤ 63 years	63.0	1		58.1	1	
> 63 years	44.5	1.66 (1.16-2.38)	0.006	52.0	1.30 (0.86-1.95)	0.20
Gender						
Male	53.6	1		52.2	1	
Female	55.9	0.90 (0.61-1.32)	0.59	62.4	0.90 (0.58-1.39)	0.64
Clinical stage (7^th^ ed.)						
I + II	63.8	1		59.9	1	
III + IV	45.0	1.68 (1.17-2.41)	0.005	50.5	1.46 (0.97-2.19)	0.06
Location						
Tongue	55.6	1		59.6	1	
Floor of mouth	60.0	0.91 (0.54-1.54)	0.74	56.7	1.29 (0.71-2.32)	
Other	41.2	1.60 (0.77-2.21)	0.46	44.0	1.38 (0.77-2.49)	0.27
Histopathological grading						
Well-differentiated	57.9	1		60.3	1	
Moderately-differentiated	54.3	1.08 (0.73-1.61)		48.3	1.30 (0.65-2.57)	
Poorly-differentiated	44.8	1.54 (0.82-2.91)	0.30	46.6	1.37 (0.87-2.16)	0.31
Treatment						
Surgery	58.6	1		55.5	1	
Surgery + Radiotherapy	43.9	1.34 (0.88-2.03)		61.0	0.84 (0.53-1.32)	
Surgery + Radiotherapy + Chemotherapy	60.6	0.94 (0.58-1.51)	0.24	49.9	1.34 (0.78-2.28)	0.21
Margin status						
≥5 mm	58.2	1		56.5	1	
<5 mm	55.8	1.02 (0.64-1.57)	0.98	55.8	1.21 (0.74-1.99)	0.43
STIP1						
Low expression	66.5	1		60.1	1	
High expression	29.7	3.07 (2.04-4.64)	<0.0001	38.4	2.19 (1.36-3.53)	0.003

Taking the diagnosis of OSCC as a classification variable and the whole sample (85 controls from cohort 1, and 347 OSCCs from cohorts 1 and 2), the immunoexpression of STIP1 showed a diagnosis potential for OSCC, with an area under the ROC curve of 0.890 (95% CI: 0.832-0.934, p<0.0001) (**Supplementary Figure 5**).

### STIP1 knockdown controls proliferation, migration, invasion and metastasis

**Figure 2:**
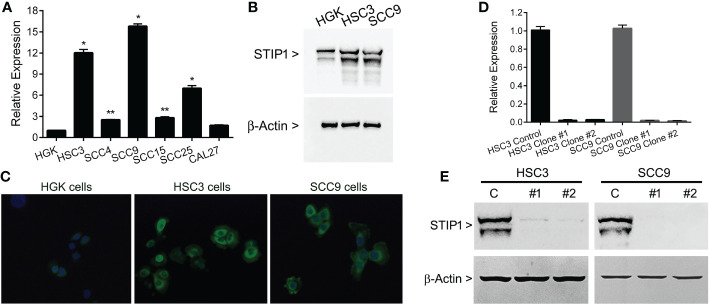
STIP1 is overexpressed in OSCC-derived cell lines. **(A)** Total RNA from the normal human gingival keratinocyte cell line (HGK) and 6 human OSCC cell lines (SCC4, SCC9, SCC15, SCC25, HSC3 and CAL27) were converted in cDNA and subjected to qPCR. The STIP1 mRNA levels were significantly higher in OSCC cells lines than in HGK, with exception of CAL27. **(B)** The overexpression of STIP1 was confirmed with western blot applying specific antibodies against STIP1 and the housekeeping control β-actin. **(C)** Immunofluorescence analysis revealed an intense staining for STIP1 in OSCC cells, which was mainly found in the perinuclear area. STIP1 knockdown efficiency in HSC3 and SCC9 cells after transduced with lentivirus expressing shRNA sequences against STIP1 (clones #1 and #2) and control as outlined in the methods. A marked reduction in both mRNA **(D)** and protein **(E)** levels when compared with control cells was observed. The values represent the average ± SD of three separate experiments. In figures **(B, C, E)** a representative image is shown. *p<0.01, **p<0.0001.

To select OSCC cell lines with high expression of STIP1, RT-qPCR was performed in a series of oral cancer cells. The levels of STIP1 mRNA were significantly higher in HSC3 (p<0.0001), SCC4 (p<0.01), SCC9 (p<0.0001), SCC15 (p<0.01) and SCC25 (p<0.0001) cells than in HGK cells (**Figure 2A**). The high levels of STIP1 in both HSC3 and SCC9 were confirmed by western blot (**Figure 2B**) and immunofluorescence (**Figure 2C**), and these cells were then selected by STIP1 knockdown. As expected, cells transduced with lentivirus carrying the specific shRNA sequences targeted to the STIP1 transcript demonstrated a significant reduction in both STIP1 mRNA (**Figure 2D**) and protein (**Figure 2E**) levels in comparison with cells transduced with the non-targeting sequence (control).

Cell viability and apoptotic rates were not altered after STIP1 silencing (**Supplementary Figure 6**). However, when the cells were exposed to hydrogen peroxide to promote oxidative stress-induced cell death, a significant reduction in cell viability in STIP1-silencing clones for both HSC3 and SCC9 cells was detected (**Supplementary Figure 6**). STIP1 knockdown did not influence the sensibility of the cells to cisplatin treatment (**Supplementary Figure 6**).

**Figure 3:**
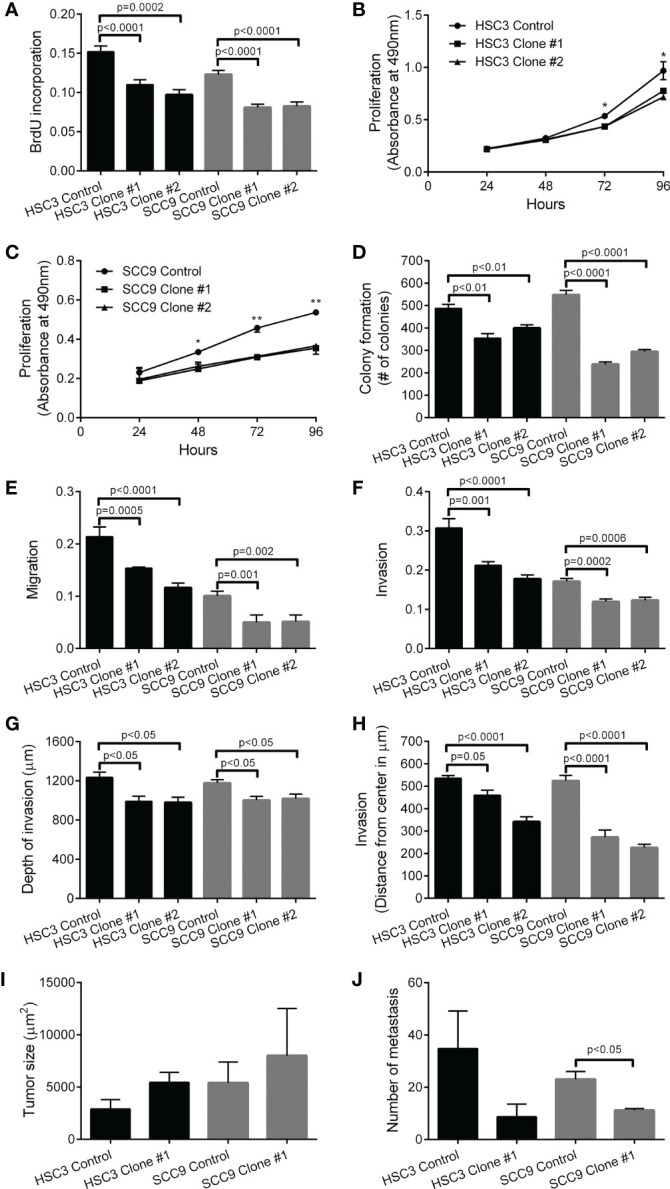
Knockdown of STIP1 controls proliferation, migration, invasion and metastasis. Downregulation of STIP1 significantly inhibited the proliferative potential of HSC3 and SCC9 cells, as revealed BrdU incorporation index **(A)**, growth curves **(B, C)** and number of colonies in the colony formation assay **(D)**. Migration **(E)** and Invasion **(F)** of HSC3 and SCC9 cells were significantly decreased by STIP1-specific shRNAs, as revealed by transwell-based assays. The impaired invasion of both HSC3 and SCC9 cells after STIP1 knockdown was observed in the organotypic myoma assay **(G)**, represented by low depth of invasion, and in the 3D spheroid assays **(H)**. In the zebrafish model, no significant differences in volume of tumors formed by HSC3 and SCC9 cells with STIP1 knockdown or controls were observed **(I)**, but the number of metastatic foci were decreased in STIP1 silenced cells, reaching significant levels for SCC9 cells **(J)**. The values represent the average ± SD of three separate experiments. In the organotypic myoma assay, three deepest islands were measured in four microscopic fields of six myomas. In a zebrafish model, n=16-21 in three independent experiments. *p<0.05, **p<0.001.

Reduced levels of STIP1 significantly decreased HSC3 and SCC9 proliferation as revealed by BrdU incorporation assay (**Figure 3A**), growth curves (**Figures 3B, C**) and colony formation assay (**Figure 3D**). Repression of STIP1 expression significantly inhibited migration (**Figure 3E**) and invasion of OSCC cells, as depicted in the transwell invasion assay (**Figure 3F**). This invasion effect was also verified in the organotypic myoma (**Figure 3G**) and the 3D spheroid assays (**Figure 3H**) as reduced depth/distance of invasion. In the myoma assay, invasion area and island size were significantly smaller in both STIP1-silenced SCC9 cells than in the control cells (**Supplementary Figure 7**). The impacts of STIP1 silencing on tumor growth and metastasis *in vivo* were explored with HSC3 clone #1 and SCC9 clones #1 in a zebrafish model (**Supplementary Figure 8**). The sizes of primary tumors were slightly larger; however, the difference was not statistically significant (**Figure 3I**). The number of metastases was obviously lower in STIP1 silenced cells, reaching significance for SCC9 cells (**Figure 3J**).

### STIP1 is regulated by miR-218-5p in OSCCs

**Figure 4:**
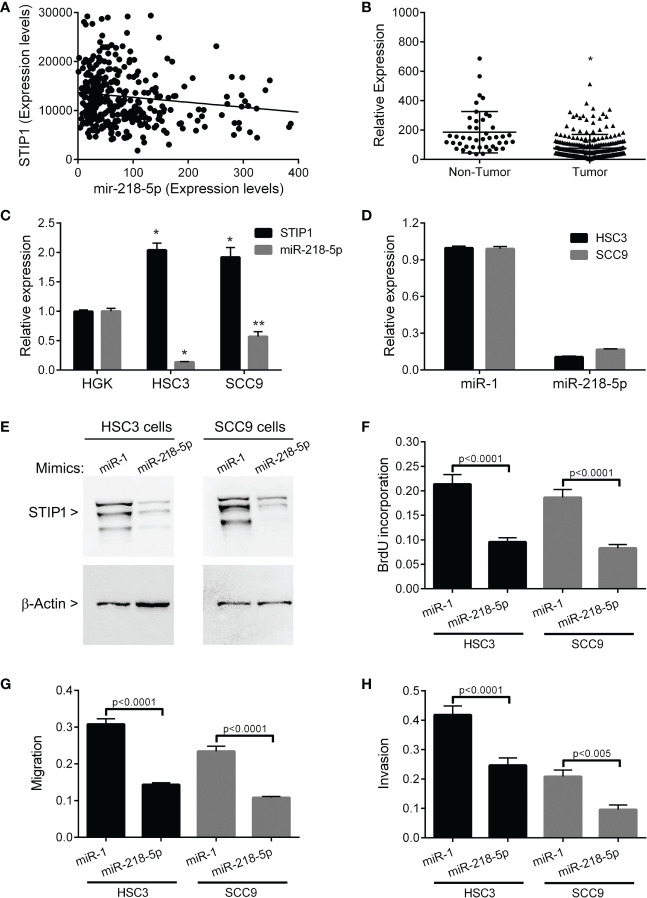
STIP1 and miR-218-5p in OSCC. **(A)** Expression of STIP1 is inversely correlated with miR-218-5p in OSCC samples from TCGA (rs=0.216, p=0.02). **(B)** The levels of miR-218-5p are down-regulated in OSCCs compared to control (non-tumor samples) (*p<0.0001). **(C)** There is a clear inverse correlation between STIP1 and miR-218-5p in the HSC3 and SCC9 cell lines (*p<0.0001, **p<0.005). Levels of STIP1 mRNA **(D)** and protein **(E)** were clearly decreased after transfection of miR-218-5p mimics, demonstrating that miR-218-5p regulates STIP1 mRNA levels. Forced overexpression of miR-218-5p significantly inhibited the proliferation **(F)**, migration **(G)** and invasion **(H)** of HSC3 and SCC9 cells. The values represent the average ± SD of three separate experiments. In figure **(E)** a representative image is shown.

In the OSCC sample of the TCGA database, miR-218-5p was identified as one of the STIP1 inversely correlated microRNAs (rs=0.216, p=0.02, **Figure 4A**), and in silico target analyses revealed a conserved miR-138-5p-binding site within STIP1 mRNA. Compared with non-tumor samples, miR-218-5p showed significantly lower expression level in OSCCs (p<0.0001, **Figure 4B**), and its level was significantly higher in HGK than in HSC3 (p<0.0001) and SCC9 (p<0.005) cells (**Figure 4C**). In both HSC3 and SCC9 cell lines, STIP1 expression was inversely correlated with miR-218-5p. HSC3 and SCC9 cells transfected with miR-218-5p mimics resulted in a clear decrease in STIP1 mRNA (**Figure 4D**) and protein (**Figure 4E**). Up-regulation of miR-218-5p also restrained proliferation (**Figure 4F**), migration (**Figure 4G**) and invasion (**Figure 4H**) ability of HSC3 and SCC9 cells.

## Discussion

OSCC is frequently life-threatening, especially if not diagnosed and treated early. Contributing to this poor behavior are its intrinsic aggressiveness with an increased tendency to invasion and metastasis, the diagnosis frequently at advanced stage, and the lack of robust indicators for clinical applications, such as responsiveness to treatment, therapeutic targets, prognosis and post-therapeutic monitoring (34). Thereafter, it is essential to identify reliable biomarkers to effectively assess the aggressiveness of tumors and choose the optimal therapeutic strategy, making OSCC treatment more effective and predictable.

STIP1 is described as playing a significant role in tumor progression. A recent meta-analysis suggests that high STIP1 expression is significantly associated with shorter overall survival, early lymph node metastasis and more advanced clinical stage compared with low STIP1 expression in six studied cancer types (35). To date, the expression of STIP1 in OSCC tissues and its association with clinical features and survival of the patient have not been examined. Therefore, the aim of this study was to quantify STIP1 in OSCCs samples compared to non-tumor tissues and evaluate its prognostic value. We also studied the effects of STIP1 silencing on OSCC cell behavior and revealed a microRNA molecule regulating STIP1.

This study shows that STIP1 is a prognostic biomarker for OSCC. STIP1 is overexpressed at both mRNA and protein levels in OSCC samples compared with normal tissues, similar as, for example, in gliomas (13), colorectal cancers (15), hepatocellular carcinoma (18), gastric cancers (20), cervical carcinomas (21), lung cancers (23), pancreatic cancers (36) and breast cancers (37). Furthermore, our findings demonstrate that higher levels of STIP1 were significantly and independently associated with reduced CSS and poor DFS compared with low STIP1 expression. These findings are in line with previous studied with other cancers showing poor overall survival and/or advanced cancer stage (15, 19, 23, 36–38). Supported by the results with other cancers, our data suggest that STIP1 overexpression is an independent prognostic marker associated with OSCC patient outcome.

The strategy of reducing STIP1 levels has been successfully applied in several cancer cell lines. In the current study, silencing of STIP1 impaired proliferation, migration and invasion ability of OSCC cell lines *in vitro*, while cell viability and apoptotic rate were not affected. However, under hydrogen peroxide-induced oxidative stress, the viability of the STIP1 knockdown cells was significantly reduced, supporting a protective effect under specific conditions. These STIP1 effects have been reported in a number of physiological and pathophysiological conditions. For example, STIP1 knockdown also inhibited proliferation and motility of colorectal (15), breast (19), lung (23), glioma (13), and gastric (20) cancer cell lines, emphasizing that high expression of STIP1 is linked to cancer progression. In cervical carcinoma (21), breast cancer (19), and lung cancer (23) cells, STIP1 knockdown induced apoptosis without any stress condition, which differs from our results with OSCC cells and suggests differential regulation of apoptotic process by STIP1, which can be related to the observed dysregulation of the autocrine effects of cytokines and/or growth factors. In the zebrafish model, the size of primary tumors was slightly larger, however, silenced cells were less metastatic than control cells. In mouse metastatic models with STIP1 silenced in gastric cancer cells (20) and in hepatocellular carcinoma cells (39), metastatic lung nodules were inhibited. Taken together, we suggest that proliferation, motility and metastatic ability of OSCC cells are related to STIP1 overexpression, which induces a more aggressive behavior, as observed in the cohorts of patients with OSCC. Although we were unable to connect STIP1 overexpression and response to cisplatin, it was previously reported that STIP1 can be a predictor of cisplatin-resistance in bladder cancer patients, which are candidates to immunotherapy (40). Further studies exploring STIP1 overexpression and chemoresistance, and STIP1 levels with PD1 or CTLA4 could be interesting.

The studies reported here also revealed that STIP1 and miR-218-5p are inversely correlated, and the transfection of OSCC cell lines with miR-218-5p mimics downregulated STIP1 which was followed by inhibition of proliferation, motility and invasiveness, similarly as seen with STIP1 silencing. Recently, miR-218-5p was reported to be downregulated in several cancers, including gastric, colorectal, ovarian, prostate, cervical and lung adenocarcinoma (41–46) and have different protein targets affecting cancer progression. Downregulation of miR-218-5p promoted OSCC invasion *via* activating CD44-ROCK signaling (47). A recent study demonstrated the relationship between long non-coding RNA HOXA-AS3 and miR-218-5p (48). While HOXA-AS3 is upregulated in OSCCs, miR-218-5p is downregulated in comparison with para-cancerous tissues. Moreover, HOXA-AS3 impaired proliferation induced by forced overexpression of miR-218-5p. Nevertheless, our study has an important limitation, because we did not carry out assays to demonstrate that STIP1 is a direct target of miR-218-5p. Thereafter, the interaction and regulation between miR-218-5p and STIP1 expression should be further investigated.

In the last decade, the adoption of many targeted drugs for cancer therapy has improved the outcome of patients with different types of cancer. However, the advances are more limited for oral cancer. Cetuximab, an antibody targeting the extracellular domain of epidermal growth factor receptor (EGFR), applied in combination with radiotherapy for advanced or recurrent cases, is the only targeted-drug approved for OSCC (49). In this sense, the identification of new potential targets, such as STIP1, is essential and urgent. However, it is important to consider important limitations, including an effective delivery system, because most STIP1 is found in the cytosol in intime contact with the Golgi complex, and different grades of toxicity due to disruption of normal cellular function are expected, because STIP1 is also expressed in normal cells.

Although heat-shock proteins and their associated proteins are not mutated in cancers, they are traditionally overexpressed as an adaptive mechanism to counteract the adverse effects and to bring advantages to the tumor. Here, we demonstrate that STIP1 is overexpressed in OSCCs, which is at least in part caused by downregulation of miR-218-5p, and its high levels are significantly associated with shorter survival and high risk for recurrence. Moreover, the *in vitro* and *in vivo* results confirmed that high STIP1 expression affects proliferation, migration, invasion and metastatic potential of the OSCC cells. A better understanding and knowledge of the biological events of specific players in OSCCs, such as STIP1, and following studies precede the clinical adoption of a biomarker, which can improve the treatment and increase the survival rates of patients with OSCC.

## Data availability statement

The original contributions presented in the study are included in the article/**Supplementary Material**. Further inquiries can be directed to the corresponding author.

## Ethics statement

The studies involving human participants were reviewed and approved by Approval from the ethics review board of each of the hospitals affiliated with the collaborative study was obtained, and the study was approved by Human Research Ethics Committee of the School of Dentistry, University of Campinas (CAAE: 55927322.0.0000.5418). Written informed consent was obtained from participants in compliance with the World Medical Association Declaration of Helsinki, Ethical Principles for Medical Research Involving Human Subjects. The patients/participants provided their written informed consent to participate in this study. The animal study was reviewed and approved by University of Helsinki under the ethical permission (ESAVI/13139/04.10.05/2017) given by the regional state administrative agency.

## Author contributions

All authors have made a substantial, direct and intellectual contribution to the work and approved it for publication.

## Supplementary material

Supplementary Figure 1 Immunohistochemistry analysis of STIP1 performed in the tissues disposed of in a tissue microarray (cohort 1). Representative images of the STIP1 immunostaining in a normal mucosa tissue **(A)**, primary OSCC **(B)** and lymph node metastasis **(C)**. (Original magnification x100) **(D)** Quantification of the staining revealed a significant higher expression of STIP1 in both primary tumors and lymph node metastases compared to normal mucosa tissues. *p<0.0001

Supplementary Figure 2 Kaplan-Meier survival curves for patients of cohort 1. **(A)** Cancer-specific survival and **(B)** disease-free survival.

Supplementary Figure 3 Representative immunohistochemical images of the STIP1 stain in the whole sections belonging to cohort 2. A clear cytoplasmic staining for STIP1 was observed, which was quite similar to that observed in the cohort 1. **(A)** Representative samples classified with low STIP1 expression and **(B)** representative sample with high expression of STIP1. (Original magnification x200)

Supplementary Figure 4 Expression of STIP1 is associated with shortened survival of patients with OSCC. The cancer-specific survival **(A)** and disease-free survival **(B)** according to the Kaplan-Meier method.

Supplementary Figure 5 Receiver operating characteristic (ROC) curve with area under the curve (AUC) comparing STIP1 staining between OSCC samples and normal tissues (non-tumor samples). The diagnostic potential of STIP1 is clearly observed.

Supplementary Figure 6 Downregulation of STIP1 affects viability of HSC3 and SCC9 cells just under stress conditions. Knockdown of STIP1 did not affect the viability **(A)** and apoptotic rate **(B)** of the cells cultured under normal conditions. **(C)** A significant reduction in the viability of the HSC3 and SCC9 silenced cells was observed after treatment with 125 µM hydrogen peroxide for 1 h. **(D, E)** The knockdown of STIP1 did not modulate the sensibility of the cells to cisplatin. The values represent the average ± SD of three separate experiments.

Supplementary Figure 7 Quantification of the invasion area **(A)** and island size **(B)** in the myoma organotypic model for 14 days. The invasion area and island size were significantly reduced in SCC9 knockdown cells compared to control, but no effects on invasion area was observed HSC3 cell. Interesting, the island sizes were slightly higher in HSC3 clones #1 and #2 compared to control HSC3 cells. The values represent the average ± SD of six myomas each analyzed from four microscopic fields.

Supplementary Figure 8 Representative images of the Zebrafish xenograft model. Tumor cells (red) were xenotransplanted into two day-old fishes, and tumor area (arrow) and number of metastatic foci (arrowhead) were determined after 72 h. Representative fishes xenotransplanted with HSC3 control **(A)**, HSC3 clone #1 **(B)**, SCC9 control **(C)** and SCC9 clone #1 **(D)** are shown.
