# Supplementary material for: Stress induced phosphoprotein 1 overexpression controls proliferation, migration and invasion and is associated with poor survival in oral squamous cell carcinoma
Source: Front Oncol. 2023 Jan 11;12:1085917. doi: 10.3389/fonc.2022.1085917 (PMC9874128; doi:10.3389/fonc.2022.1085917)
Supplement: Supplementary file 1 [file Table_1.docx]

Supplementary Table 1. Clinicopathological features of patients with oral squamous cell carcinoma from cohort 1 (TMA, n=85) and cohort 2 (whole sections, n=262).

|  | Cohort 1 | Cohort 2 |
| --- | --- | --- |
| Age (years) |  |  |
| Mean ± SD | 64.1 ± 13.7 | 62.7 ± 14.1 |
| Range | 32-98 | 17-99 |
| Sex |  |  |
| Male | 47 (55.3%) | 181 (69.1%) |
| Female | 38 (44.7%) | 81 (30.9%) |
| Clinical stage |  |  |
| I | 22 (25.9%) | 50 (19.1%) |
| II | 16 (18.8%) | 57 (21.7%) |
| III | 14 (16.5%) | 47 (17.9%) |
| IV | 28 (32.9%) | 60 (22.9%) |
| Missing data | 5 (5.9%) | 48 (18.4%) |
| Location |  |  |
| Tongue | 55 (64.7%) | 185 (70.6%) |
| Floor of month | 8 (9.4%) | 34 (11.8%) |
| Others | 22 (25.9%) | 42 (16.0%) |
| Missing | - | 1 (1.6%) |
| Histopathological grading |  |  |
| Well-differentiated | 12 (14.1%) | 78 (29.8%) |
| Moderately-differentiated | 50 (58.8%) | 152 (58.0%) |
| Poorly-differentiated | 21 (24.7%) | 32 (12.2%) |
| Missing data | 2 (2.4%) | - |
| Treatment |  |  |
| Surgery | 59 (69.4%) | 121 (46.2%) |
| Surgery + Radiotherapy | 20 (23.5%) | 85 (32.4%) |
| Surgery + Radiotherapy + Chemotherapy | 6 (7.1%) | 51 (19.4%) |
| Missing data | - | 5 (2.0%) |
| Margin status |  |  |
| ≥5 mm | 67 (78.8%) | 169 (64.5%) |
| <5 mm | 16 (18.8%) | 64 (24.4%) |
| Missing data | 2 (2.4%) | 29 (11.1%) |
| Recurrence |  |  |
| No | 26 (30.6%) | 148 (56.5%) |
| Yes | 41 (48.2%) | 98 (37.4%) |
| Missing data | 18 (21.2%) | 16 (6.1%) |
| Status |  |  |
| Alive | 51 (60.0%) | 140 (53.4%) |
| Dead | 34 (40.0%) | 122 (46.6%) |
