# Supplementary material for: Stress induced phosphoprotein 1 overexpression controls proliferation, migration and invasion and is associated with poor survival in oral squamous cell carcinoma
Source: Front Oncol. 2023 Jan 11;12:1085917. doi: 10.3389/fonc.2022.1085917 (PMC9874128; doi:10.3389/fonc.2022.1085917)
Supplement: Supplementary file 2 [file Table_2.docx]

Supplementary Table 2. Association of STIP1 expression and the clinicopathological characteristics of the patients in the cohort 1.

| Parameter | Low STIP1  n (%) | High STIP1  n (%) | p value |
| --- | --- | --- | --- |
| Age |  |  |  |
| ≤ 63 years | 21 (47.7) | 18 (43.9) |  |
| > 63 years | 23 (52.3) | 23 (56.1) | 0.72 |
| Gender |  |  |  |
| Male | 24 (54.5) | 23 (56.1) |  |
| Female | 20 (45.5) | 18 (43.9) | 0.88 |
| Clinical stage (7^th^ ed.) |  |  |  |
| Early (I + II) | 25 (59.5) | 13 (34.2) |  |
| Advanced (III + IV) | 17 (40.5) | 25 (65.8) | 0.025 |
| Tumor site |  |  |  |
| Tongue | 26 (59.1) | 29 (70.7) |  |
| Floor of mouth | 3 (6.8) | 5 (12.2) |  |
| Other | 15 (34.1) | 7 (17.1) | 0.17 |
| Histopathological grading |  |  |  |
| Well-differentiated | 9 (21.4) | 3 (7.3) |  |
| Moderately-differentiated | 23 (54.8) | 27 (65.9) |  |
| Poorly-differentiated | 10 (23.8) | 11 (26.8) | 0.18 |
| Treatment |  |  |  |
| Surgery | 30 (68.2) | 29 (70.7) |  |
| Surgery + Radiotherapy | 10 (22.7) | 10 (24.4) |  |
| Surgery + Radiotherapy + Chemotherapy | 4 (9.1) | 2 (4.9) | 0.75 |
| Margin status |  |  |  |
| ≥5 mm | 34 (81.0) | 33 (80.5) |  |
| <5 mm | 8 (19.0) | 8 (19.5) | 0.97 |
| Recurrence |  |  |  |
| No | 17 (47.2) | 9 (29.0) |  |
| Yes | 19 (52.8) | 22 (71.0) | 0.13 |
