# Supplementary material for: Stress induced phosphoprotein 1 overexpression controls proliferation, migration and invasion and is associated with poor survival in oral squamous cell carcinoma
Source: Front Oncol. 2023 Jan 11;12:1085917. doi: 10.3389/fonc.2022.1085917 (PMC9874128; doi:10.3389/fonc.2022.1085917)
Supplement: Supplementary file 3 [file Table_3.docx]

Supplementary Table 3. Association of the clinicopathological features of tumors (cohort 2) with STIP1 expression.

| Parameter | Low STIP1  n (%) | High STIP1  n (%) | p value |
| --- | --- | --- | --- |
| Age |  |  |  |
| ≤ 63 years | 78 (42.9) | 48 (64.9) |  |
| > 63 years | 104 (57.1) | 26 (35.1) | 0.0014 |
| Gender |  |  |  |
| Male | 126 (69.2) | 53 (71.6) |  |
| Female | 56 (30.8) | 21 (28.4) | 0.70 |
| Clinical stage (7^th^ ed.) |  |  |  |
| Early (I + II) | 99 (55.0) | 28 (38.4) |  |
| Advanced (III + IV) | 81 (45.0) | 45 (61.6) | 0.017 |
| Tumor site |  |  |  |
| Tongue | 138 (76.2) | 43 (58.1) |  |
| Floor of mouth | 17 (9.4) | 15 (20.3) |  |
| Other | 26 (14.4) | 16 (21.6) | 0.01 |
| Histopathological grading |  |  |  |
| Well-differentiated | 55 (30.2) | 21 (28.4) |  |
| Moderately-differentiated | 105 (57.7) | 43 (58.1) |  |
| Poorly-differentiated | 22 (12.1) | 10 (13.5) | 0.93 |
| Treatment |  |  |  |
| Surgery | 90 (50.5) | 27 (36.5) |  |
| Surgery + Radiotherapy | 56 (31.5) | 28 (37.8) |  |
| Surgery + Radiotherapy + Chemotherapy | 32 (18.0) | 19 (25.7) | 0.11 |
| Margin status |  |  |  |
| ≥5 mm | 106 (67.1) | 58 (84.1) |  |
| <5 mm | 52 (32.9) | 11 (15.9) | 0.009 |
| Recurrence |  |  |  |
| No | 113 (65.7) | 31 (44.9) |  |
| Yes | 59 (34.3) | 38 (55.1) | 0.003 |
