# Supplementary figures and images for: Stress induced phosphoprotein 1 overexpression controls proliferation, migration and invasion and is associated with poor survival in oral squamous cell carcinoma
Source: Front Oncol. 2023 Jan 11;12:1085917. doi: 10.3389/fonc.2022.1085917 (PMC9874128; doi:10.3389/fonc.2022.1085917)

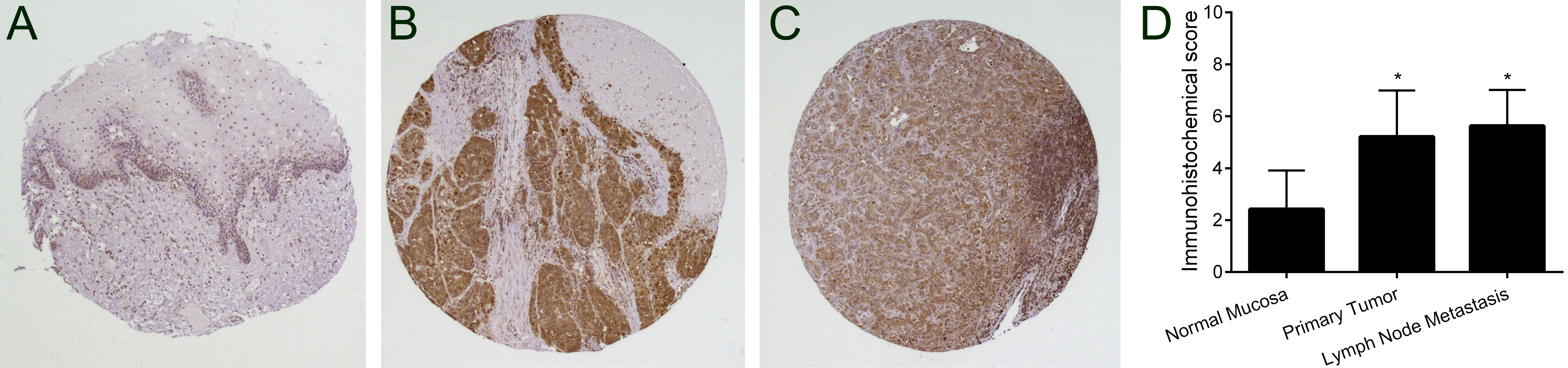

Supplement: Supplementary Figure 1 — Immunohistochemistry analysis of STIP1 performed in the tissues disposed of in a tissue microarray (cohort 1). Representative images of the STIP1 immunostaining in a normal mucosa tissue (A), primary OSCC (B) and lymph node metastasis (C). (Original magnification x100) (D) Quantification of the staining revealed a significant higher expression of STIP1 in both primary tumors and lymph node metastases compared to normal mucosa tissues. *p<0.0001 [file Image_1.jpeg]

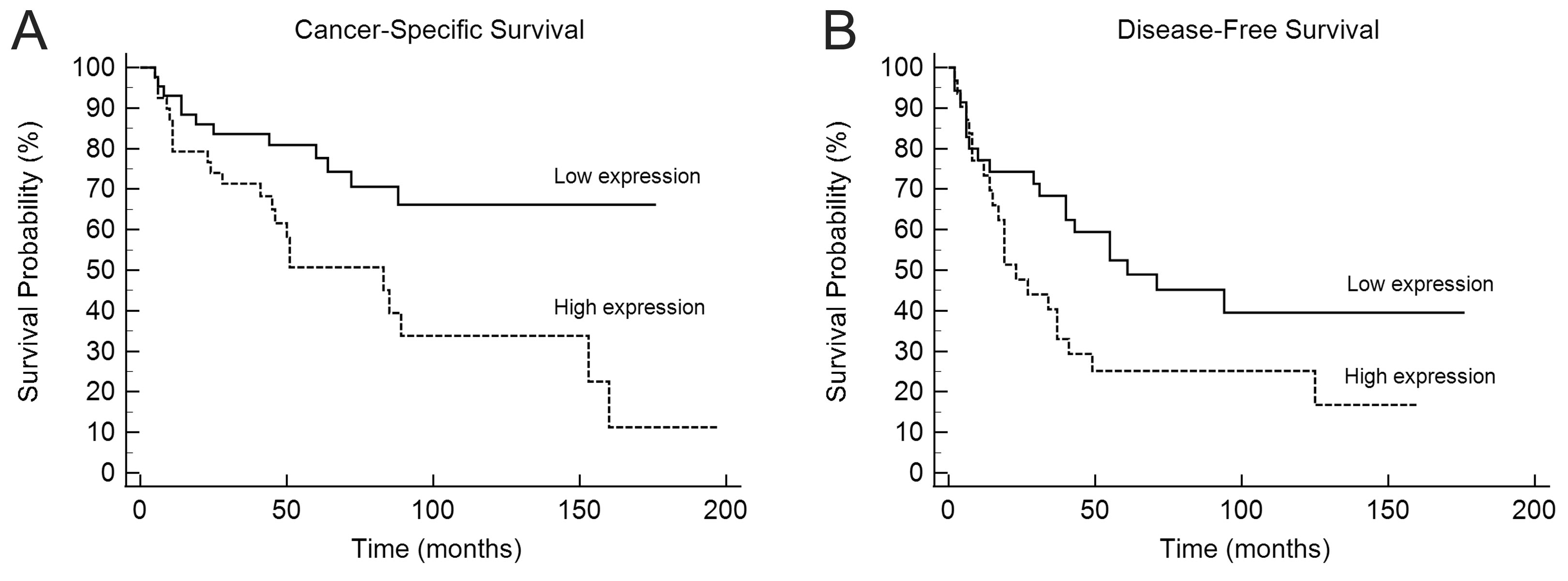

Supplement: Supplementary Figure 2 — Kaplan-Meier survival curves for patients of cohort 1. (A) Cancer-specific survival and (B) disease-free survival. [file Image_2.jpeg]

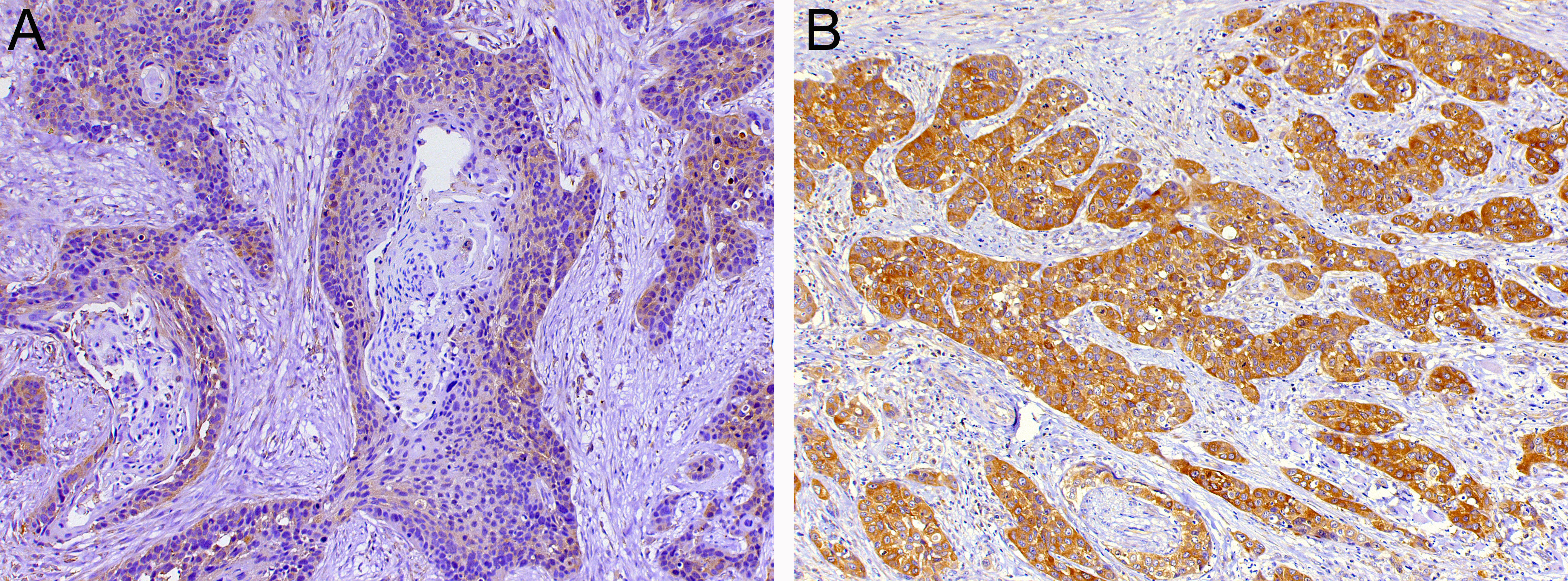

Supplement: Supplementary Figure 3 — Representative immunohistochemical images of the STIP1 stain in the whole sections belonging to cohort 2. A clear cytoplasmic staining for STIP1 was observed, which was quite similar to that observed in the cohort 1. (A) Representative samples classified with low STIP1 expression and (B) representative sample with high expression of STIP1. (Original magnification x200) [file Image_3.jpeg]

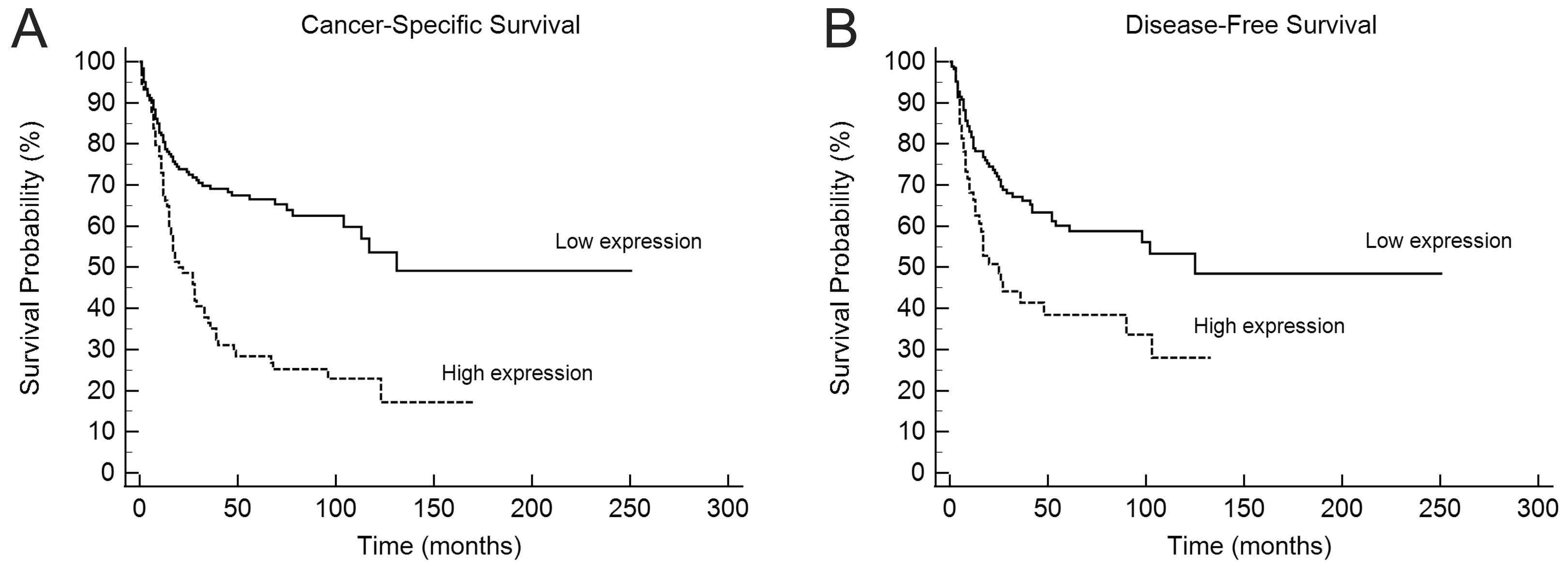

Supplement: Supplementary Figure 4 — Expression of STIP1 is associated with shortened survival of patients with OSCC. The cancer-specific survival (A) and disease-free survival (B) according to the Kaplan-Meier method. [file Image_4.jpeg]

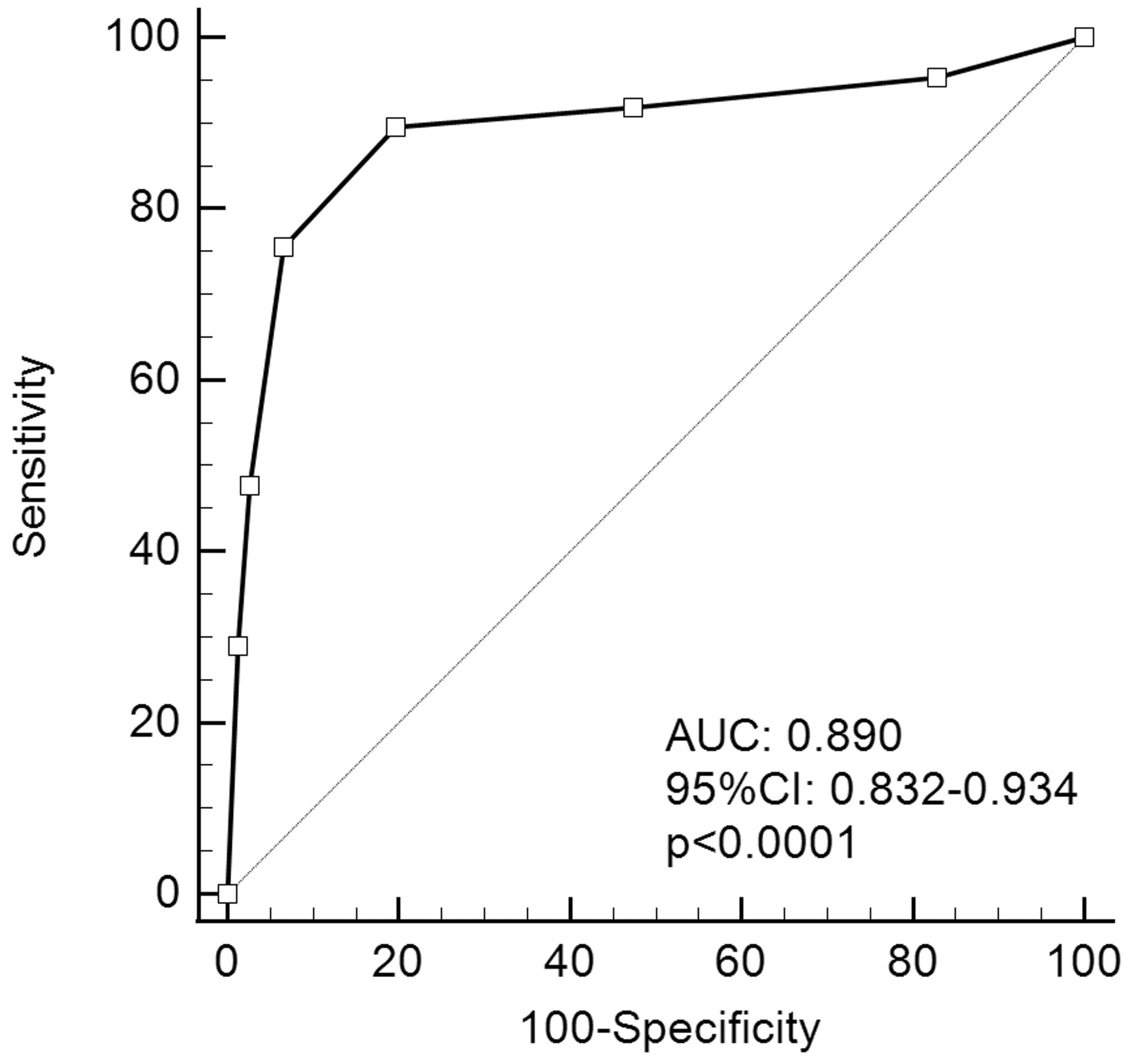

Supplement: Supplementary Figure 5 — Receiver operating characteristic (ROC) curve with area under the curve (AUC) comparing STIP1 staining between OSCC samples and normal tissues (non-tumor samples). The diagnostic potential of STIP1 is clearly observed. [file Image_5.jpeg]

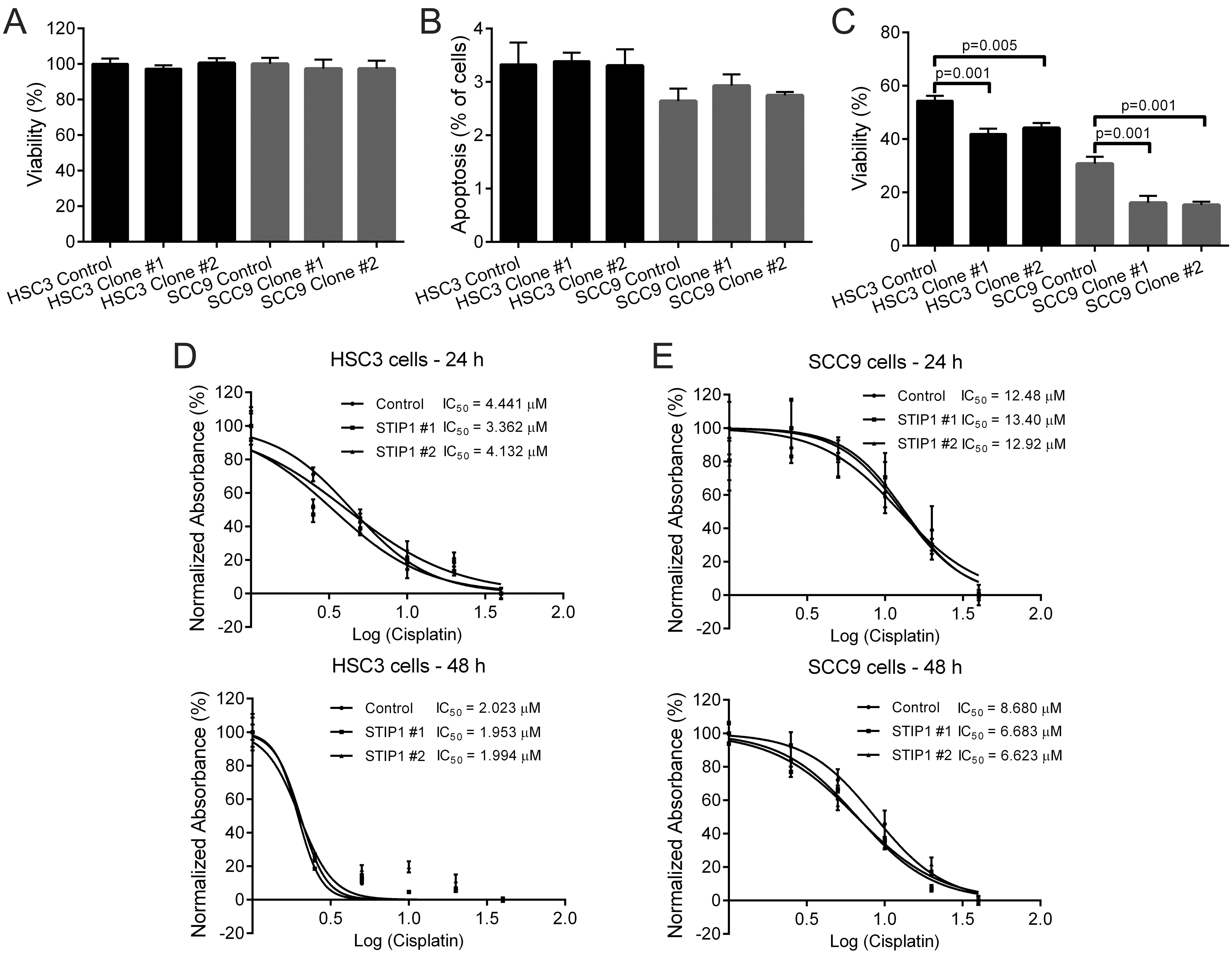

Supplement: Supplementary Figure 6 — Downregulation of STIP1 affects viability of HSC3 and SCC9 cells just under stress conditions. Knockdown of STIP1 did not affect the viability (A) and apoptotic rate (B) of the cells cultured under normal conditions. (C) A significant reduction in the viability of the HSC3 and SCC9 silenced cells was observed after treatment with 125 µM hydrogen peroxide for 1 h. (D, E) The knockdown of STIP1 did not modulate the sensibility of the cells to cisplatin. The values represent the average ± SD of three separate experiments. [file Image_6.jpeg]

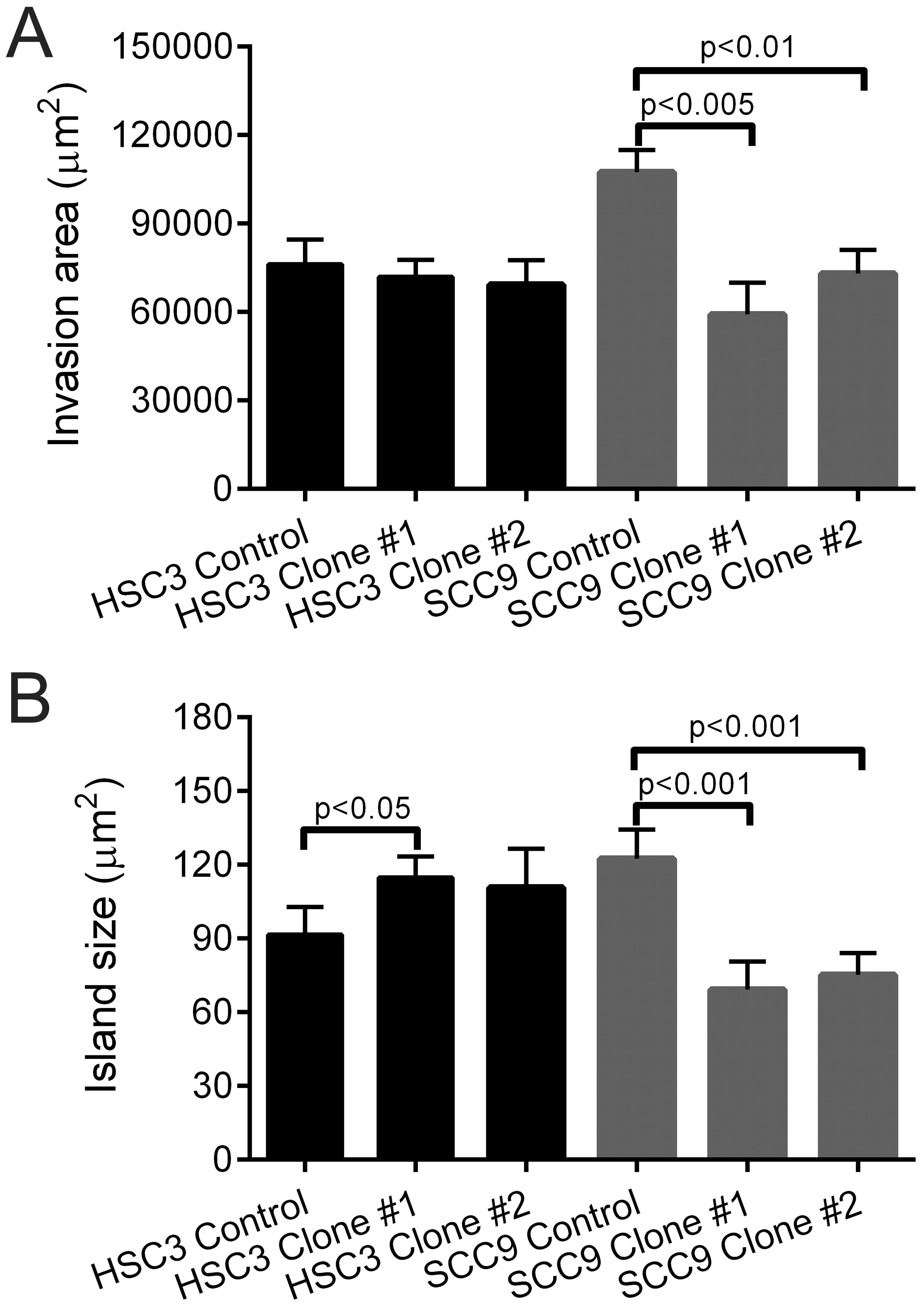

Supplement: Supplementary Figure 7 — Quantification of the invasion area (A) and island size (B) in the myoma organotypic model for 14 days. The invasion area and island size were significantly reduced in SCC9 knockdown cells compared to control, but no effects on invasion area was observed HSC3 cell. Interesting, the island sizes were slightly higher in HSC3 clones #1 and #2 compared to control HSC3 cells. The values represent the average ± SD of six myomas each analyzed from four microscopic fields. [file Image_7.jpeg]

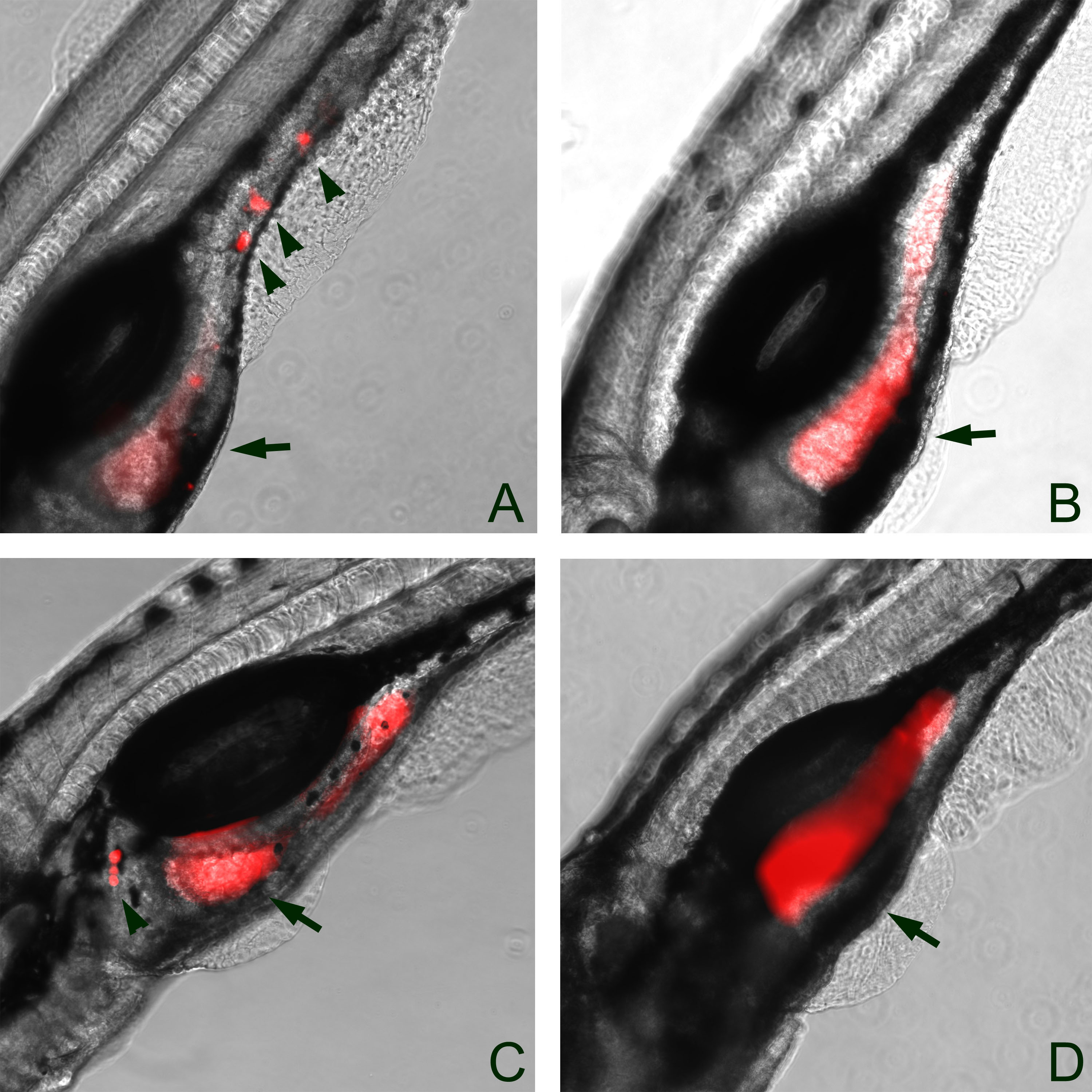

Supplement: Supplementary Figure 8 — Representative images of the Zebrafish xenograft model. Tumor cells (red) were xenotransplanted into two day-old fishes, and tumor area (arrow) and number of metastatic foci (arrowhead) were determined after 72 h. Representative fishes xenotransplanted with HSC3 control (A), HSC3 clone #1 (B), SCC9 control (C) and SCC9 clone #1 (D) are shown. [file Image_8.jpeg]
